# Hepatic Abscess

**Published:** 1886-09

**Authors:** L. L. MacArthur

**Affiliations:** Lecturer on Histology, Chicago Medical College; 2139 Wabash avenue


THE CHICAGO

MEDICAL JOURNAL AND EXAMINER.

VOL. LIII. SEPTEMBER, 1886. No. 3.

### ORIGINAL COMMUNICATIONS.

### HEPATIC ABSCESS, WITH PRESENTATION OF FOUR CASES.

*By* L. L. MACARTHUR, M. D., *Lecturer on Histology, Chicago Medical College.*

[Read before the Chicago Medical Society, August 2.]

Abscess of the liver is in our latitude a disease of extreme rarity as compared with the tropics, where the great majority of cases have been observed and studied.

This, so far as the literature of the subject is concerned, has had the effect of tincturing the writings of our present authorities in temperate latitudes with ideas perhaps applicable in other climes, but certainly, in a measure, false here.

The ascribed causes of abscess of the liver are as numerous as the writers on the subject. But in fact they may be sifted down to quite few classes. I would subdivide them first into,

I.—Intrinsic, or causes resident in the liver itself; and

II.—Extrinsic, or causes external to and independent of the gland.

To the former would belong acute congestions (said to result from heat and cold), tumors, ecchinococci, biliary calculi, or lumbrici in bile ducts.

To the latter, infective emboli, whether carried through the portal vein or hepatic artery, traumatism, alcoholics in excess, and high temperatures, surgical operations or lesions anywhere in tract of porta vena or its communications.

And here it might be of interest to state that there have been men of recognized ability who claimed that all cases not traumatic were the result of infective emboli. Of these were Frerichs, Budd and Carrington. This, however, was because their cases were ones met with in our zone, which I shall endeavor to prove are of a different type from those seen in India.

We may divide all abscesses of liver into either,

I.—The idiopathic,

II.—The infective or embolic,

III.—The traumatic, in the order of their frequency, respectively.

I.—Concerning the first variety, I would say that because of ignorance as to the causation they have been termed idiopathic. This variety is met with in the tropics; has become known through the reports of army medical officers, chiefly British, as occurring in military men, and forms the great majority of all reported cases. It is usually single, large, and, according to the opinions of medical officers there stationed (Waring, Macpherson, M'Lean, *et. al.*), to be entirely independent of traumatism or pathological lesion; but they offer no other explanation than "the climate." If I might be allowed to theorize, I would suggest a possible explanation based on the facts that:

(1) it occurs in foreigners more frequently than in natives; (2) in those formerly accustomed to rich animal diet; (3) in the indolent and luxurious rather than in those exercising and of frugal habits; (4) in those of a gouty or rheumatic diathesis.

Now it is true that anything that will decrease very largely the alkalinity of the blood will cause a precipitation of the bile; *i.e.*, the formation of biliary calculi, both in the gall bladder and in the bile ducts. In all probability the nuclei for the cystic calculi originate in the bile ducts and are carried to the bladder, there to increase by concentric deposits. Now just such are the conditions of many of the beef-eating Englishmen, and consequently the formation of biliary calculi in the bile ducts, with occlusion and consequent abscess as a result of the foreign body. In reports of some of the cases examined, the bile ducts have been thus occluded. Such calculi would be more likely to form in such a climate than here. As to this form of hepatic abscess, I can only believe it exceedingly rare in our zone.

By all odds the most frequent abscess with which we meet belongs to the second group of *infective abscesses.*

There being two channels of infection, there are two subclasses of abscesses here.

I.—Per arteriam hepaticum (the pyæmic abscess).

II.—Per portam venam.

The first variety need detain us but a brief time. They result from infective emboli, having escaped detention in the pulmonary plexus, being detained in the hepatic plexus simultaneously with other emboli in other plexuses, more particularly the renal and pulmonary. Such emboli may come from lesions of continuity in any part of the body, most frequently compound fractures. As aseptic surgery increased such cases became fewer and fewer; *i. e.*, pyæmia and pyæmic abscess of the liver are becoming rare.

II.—When we recall the fact that all the blood from the abdominal organs, intestines, colon, stomach, spleen, vermiform appendix and (partly) prostate, etc., is returned to the general circulation by way of the liver, we can readily understand how infective lesions of any of these portal radicals may permit poisonous and infective particles to reach the liver and set up there secondary inflammations.

Hence dysenteries accompanied, as they are, by ulcerations of the intestines, cause quite frequently the trouble under consideration. Indeed, so frequently is hepatic abscess complicated by dysentery that some of our eminent pathologists have sought to find here the causative relation. The four cases which I desire to present for your consideration were all complicated in this manner, and post-mortem examination in two of the cases revealed distinct ulcerations. Numerous other lesions would here suggest themselves, *e.g.*, typhoid ulcerations (Peabody), rectal ulcerations, operations about anus and rectum, and cystotomy.

III.—Thirdly, we have the traumatic abscess, this following injury of some kind to the liver substance, whether from direct or indirect violence. To the former belong cases like the one reported by McLean as following a blow over the liver; or by Otis, in which one followed a sword-thrust, or finally in a class of cases formerly thought to be dependent upon peculiar cerebral lesions from falls on vertex, but which Henry Morris explains as more probably resulting from laceration of liver-tissue by the fall, because of its own weight.

*Symptoms.*—A word as to the nervous supply of the liver. It, like the lungs, has but very little sensation, being supplied chiefly by sympathetic, left pneumogastric and right phrenic, and so inflammatory processes may go on here without much notice being taken by the patient, just as in the lung huge cavities appear almost without the knowledge, so far as pain is concerned, of the patient. So in the liver the symptoms will vary greatly according as pure liver-tissue or the peritoneum and adjacent organs are affected; *i. e.*, 1°, to the location (in the liver) of the abscess and, 2°, its size; 3°, the other tissues involved, lungs, stomach, pleura, pericardium, 4°, and its causation being of first, second or third classes. When deeply situated and small, there may be no local symptoms whatever, only those of "pus somewhere," chill, fever, sweating, etc., with general malaise, and death result from the "silent abscess" bursting. When larger they will be dependent upon the organ or organs involved in its efforts to find an exit from the body. Pleural or pulmonary and diaphragmatic symptoms if upwards; vomiting, diarrhœa, etc., if into stomach or intestines; swelling, tenderness, and œdema of abdominal wall if by forward perforation. Locally, there is generally a sense of weight, and an uneasiness on jolting, as in metritis. Tenderness and acute pain will depend upon the extent to which the peritoneum is involved. Usually there is some increase in hepatic area, and when this is present some bulging of the side. The temperature is not very high unless the peritoneum is involved considerably, when the constitutional symptoms simulate those of peritonitis. At such times there is present an important symptom, which I find but seldom mentioned in the literature on the subject—I only recall Flint's passing mention of it, and the one by which I stumbled upon and made the diagnosis in three cases,—I refer to a peritoneal friction-sound, corresponding exactly to that of pleurisy, but far beyond the pleural area. Dr. Peabody emphasizes the fact that there is usually a very marked melancholia existing. The skin is usually of a dirty, muddy appearance, but rarely icteric. Reports of urinalysis of cases state the urine is free from bile-pigment. Inability to lie on the right side may be present.

Sometimes a pain radiates up the back to the right shoulder, and is said to be of diagnostic value.

The *anatomical characters* vary according to location and cause,—in the tropical variety, single and large ; in the infective, smaller and often multiple. The pus said to be at times of a laudable character. In the cases which I examined it was always of a chocolate color, through blood-coloring and liver-detritus, and not offensive. Lining the abscess cavity, or rather floating in its contents, are long, many-branching shreds of vessels, which interfere greatly with efforts at aspiration with a needle. The general law of abscesses holds good, that they open towards the point of least resistance ; and so, if on the superior surface of liver, through the diaphragm into pleura, lung or pericardium ; if below, into the stomach, duodenum or intestine, anteriorly through the abdominal wall and posteriorly between the ribs or into the vena cava.

*Diagnosis.*—According as the symptoms are distinct and numerous, or few and general, will the diagnosis be easy or impossible. One of the cases which I will report will be of the latter character. If more careful physical examinations were made I think the frequency of post-mortem diagnosis would be greatly lessened.

Care should be taken to differentiate between pleurisy or pneumonia of right lower lobe and hepatic abscess opening in this direction. Cancer of the liver, echinococcus and inflammation of the gall-bladder, ought not to be confounded with it. In two of the cases coming under my care I completed the diagnosis by use of a carefully cleaned hypodermic syringe. This instrument in competent hands could be used with far more frequency than it is at present without any damage ; even if penetrating perfectly healthy liver-tissue it can do no serious harm, if clean.

*Prognosis.*—The mass of medical opinion, tinctured as it has been by the reports of the Indian surgeons, is that the prognosis in this affection is *decidedly* unfavorable. To this opinion I wish to take exception, at least in such cases as we are likely to meet with in our climate, for I firmly believe that, with the new era of antiseptic surgery, the verdict ought to be modified in this, as it has been in every other branch of abdominal surgery. Let me quote some of the recognized authorities:

Macpherson says, "Prognosis is generally unfavorable, yet there is a chance if the abscess finds an exit for itself." "When air gains access, the result is seldom favorable."

Henry Morris, who was selected to write the article on the subject in the *Encyclopædia of Surgery*, a work in every way abreast of the times, says, "Prognosis unfavorable—especially if infective." "Recovery may take place if the pus *finds* an exit."

The prognosis will depend upon: I.—the kind; II.—location; III.—number and size.

*Treatment.*—It has become a surgical axiom that the earlier an abscess be evacuated the better. But in the literature the opinion seems to predominate that these abscesses are an exception, and some authors even go so far as to advise non-interference. With our present knowledge of antiseptic surgery, I believe a diagnosable abscess of liver can be opened without increasing the patient's danger either by Volkmann's or Grave's method. Certainly the plan of "laisser faire," of "letting nature take its course," ought to be given up, and dependence not be placed simply upon the routine treatment by tonics, stimulants and anodynes.

Macpherson says, "When pus has once formed the time for all active treatment is passed." Then really is time for it to begin. "As soon as *fluctuation* is felt use the aspirator." Don't! Because you can't empty such an abscess, and the treatment is only palliative, and may result in killing the patient by the escape of pus into the peritoneum.

McLean, who writes the article for Reynold's "System," says, "If it be true, as I believe it is, that the most favorable route an abscess can take is through the right lung, and the most unfavorable, *i. e.*, giving least number of recoveries, is through the abdominal wall, it appears to me unjustifiable to expose the patient by an operation to the risk of admitting *air* with all its mischievous properties into the cavity of an abscess." Here, in the light of our advances in surgery, need we fear the mischievous consequences of a little air?

Or again, Dr. Lowe, of the Madras army, than whom few have had a larger experience with this affection, says:

"Nothing can be more different from another than the method of the surgeon on the one hand and nature on the other. The surgeon discharges the whole contents of the abscess at once, *air* (awful thing) takes the place of the pus, and however great the relief, the result is disastrous. Decomposition sets in, in the discharges of the cavity, unhealthy extension of the suppurative process, and the patient succumbs.

"I have attempted to imitate nature, but all in vain; air finally entered and the result was fatal."

I have to present four cases of liver abscess, three of which occurred during my interneship at Cook County Hospital, and the other at St. Luke's Hospital.

In two of the Cook County Hospital cases operative procedures were instituted by Volkmann's method, and recovered; in the third, the expectant treatment was adopted, and death resulted; in the fourth, a diagnosis was not made, because of absence of characteristic symptoms.

2139 Wabash avenue.

